# Association between geriatric nutritional risk index and stroke prevalence in elderly adults: a cross-sectional analysis of NHANES 1999–2018

**DOI:** 10.1186/s12877-025-06959-6

**Published:** 2026-03-25

**Authors:** Chunqi Wang, Dong Zhou, Shuangyan Tu, Jing Chen

**Affiliations:** 1 Department of Neurology, West China Hospital, Sichuan University, No. 37, Guoxue Road, Wuhou District, Chengdu, Sichuan Province 610000 China; 2 West China School of Nursing, Sichuan University, Chengdu, China

**Keywords:** GNRI, stroke, elderly adults, cross-sectional, NHANES

## Abstract

**Background:**

Stroke remains a leading cause of mortality and disability worldwide, with nutritional status emerging as a crucial yet underexplored risk factor in elderly populations. The Geriatric Nutritional Risk Index (GNRI) represents a valuable nutritional assessment tool specifically developed for geriatric populations. This study examined the association between GNRI and stroke prevalence among elderly individuals using nationally representative data.

**Methods:**

This cross-sectional analysis utilized National Health and Nutrition Examination Survey (NHANES) data from 1999 to 2018, including 16,092 participants aged ≥ 60 years. GNRI was calculated using serum albumin levels and body weight ratio, with participants categorized into quartiles. Stroke status was determined through self-reported physician diagnosis. Survey-weighted logistic regression models were constructed with progressive adjustments for demographic, lifestyle, and clinical factors.

**Results:**

Among participants (mean age 70.0 years), 12.31% reported stroke history. GNRI demonstrated significant inverse association with stroke prevalence. Each one-standard-deviation increase in GNRI was associated with 12% lower stroke odds (odds ratio [OR]: 0.88; 95% CI: 0.83–0.93). Quartile analysis revealed progressively lower odds compared to the lowest quartile: Q2 (OR: 0.81; 95% CI: 0.69–0.95), Q3 (OR: 0.74; 95% CI: 0.63–0.87), and Q4 (OR: 0.77; 95% CI: 0.65–0.91) (P for trend < 0.05). Multiple analytical approaches consistently demonstrated a linear inverse association. Subgroup analyses revealed a stronger inverse association in females (OR: 0.96; 95% CI: 0.95–0.98) versus males (OR: 0.99; 95% CI: 0.97–1.00) and among current drinkers (OR: 0.96; 95% CI: 0.94–0.97).

**Conclusions:**

Higher GNRI scores were significantly associated with lower stroke prevalence in elderly adults in a linear dose-response manner, with the association being particularly pronounced in females and current drinkers. These cross-sectional findings suggest that GNRI may be a useful nutritional risk screening tool in geriatric populations; however, prospective studies are needed to establish temporality and causality.

**Supplementary Information:**

The online version contains supplementary material available at 10.1186/s12877-025-06959-6.

## Introduction

Stroke remains a leading cause of mortality and disability worldwide, with its burden increasing significantly among older populations. In the United States, stroke is the fifth leading cause of death, with an overall prevalence of 2.6% in those over 20 years of age between 2009 and 2012 [1]. In 2021, the global age-standardized incidence rate (ASIR) of ischemic stroke was 92.4 per 100,000 people, with the highest incidence observed among individuals aged 50 and older, particularly those over 60[2]. This age-related trend underscores the impact of aging on stroke risk and highlights the critical need to identify modifiable risk factors that may be associated with stroke prevalence in elderly populations. Established modifiable risk factors include hypertension, diabetes mellitus, dyslipidemia, smoking, and physical inactivity [3]. Among these factors, nutritional status has emerged as a crucial, yet often overlooked, determinant of stroke risk, especially in older adults who are more susceptible to malnutrition and its associated complications [4, 5]. 

The Geriatric Nutritional Risk Index (GNRI), developed by Bouillanne et al. in 2005, represents a particularly valuable tool for nutritional risk assessment in elderly populations due to several unique advantages over other nutritional indices [6]. Unlike single-parameter tools that rely solely on laboratory values or anthropometric measurements, GNRI incorporates both serum albumin levels and the ratio of actual to ideal body weight, providing a comprehensive evaluation of nutritional risk through its dual-parameter approach that considers both biochemical and anthropometric parameters [7, 8]. This combination is especially relevant for elderly individuals, as it captures both protein-energy malnutrition (reflected by low albumin) and weight loss (reflected by low weight-to-height ratio), two critical components of age-related nutritional decline. Furthermore, GNRI was specifically developed and validated for geriatric populations, making it more appropriate for assessing nutritional risk in relation to stroke prevalence compared to general nutritional indices that may not capture age-specific nutritional vulnerabilities.

The relationship between nutritional status and stroke risk is complex and multifaceted. Poor nutritional status has been linked to stroke risk through several pathways, including increased inflammation, endothelial dysfunction, accelerated atherosclerosis, and impaired immune function [9–11]. Malnutrition has been associated with elevated levels of inflammatory markers, arterial calcification, and atherosclerosis progression, all of which may increase stroke susceptibility. Conversely, adequate nutritional status is thought to support vascular health through antioxidant functions, optimal protein synthesis, and maintenance of vascular integrity [12–14]. 

Importantly, several prospective studies have demonstrated associations between GNRI and subsequent cardiovascular events, suggesting its potential value in risk assessment [15, 16]. It is crucial to distinguish between studies examining GNRI as a predictor of stroke risk versus those evaluating GNRI as a prognostic indicator in patients who have already experienced stroke. The vast majority of existing GNRI-stroke literature focuses on the latter, investigating how nutritional risk influences post-stroke outcomes such as functional recovery, neurological improvement, mortality, and complications in stroke patients [17–21]. In contrast, studies directly examining GNRI as a predictor of incident stroke events are remarkably limited. For instance, a large prospective cohort study of 5,312 elderly hypertensive patients followed for an average of 3.8 years found that lower GNRI values were independently associated with higher risk of incident stroke events [22]. Similarly, a 10-year follow-up study of hemodialysis patients demonstrated that lower GNRI was an independent risk factor for both brain infarction and hemorrhage [23]. These longitudinal findings from prospective studies suggest that baseline GNRI may be associated with subsequent stroke risk, indicating that the cross-sectional associations observed in our study could reflect relationships that extend beyond post-stroke nutritional decline alone.

However, significant gaps remain in our understanding of the GNRI-stroke relationship. Existing studies have primarily focused on specific clinical populations with relatively small sample sizes, limiting generalizability to the broader elderly population [22, 23]. Additionally, while previous research has demonstrated associations between nutritional indices and health outcomes, the consistency and magnitude of the GNRI-stroke relationship across diverse elderly populations have not been systematically investigated [24, 25]. Understanding the nature of the cross-sectional association between GNRI and stroke prevalence across different population subgroups is crucial, as it could help identify elderly populations who may benefit most from nutritional risk screening[26], explain why nutritional interventions show varying effectiveness across different patient groups[27], and provide foundational evidence for determining optimal GNRI targets in future intervention studies [28]. 

Therefore, this study aims to examine the association between GNRI and stroke prevalence among elderly individuals using a large, nationally representative dataset from NHANES (1999–2018), exploring potential effect modifications across different demographic and clinical subgroups and the dose-response relationship across the full GNRI spectrum using multiple analytical approaches.

## Methods

### Study population

This cross-sectional analysis utilized data from the National Health and Nutrition Examination Survey (NHANES), which was conducted between 1999 and 2018 [29]. NHANES employs a multistage, complex probability sampling strategy designed to produce a nationally representative sample of the U.S. civilian, non-institutionalized population. Initially, 101,316 individuals were included in the cohort; however, the analysis focused on adults aged 60 years and older. Participants with missing stroke data or missing components for any GNRI calculation (serum albumin, weight, or height) were excluded. Ultimately, the analytical sample consisted of 16,092 participants.

All GNRI components (serum albumin, height, and weight) and covariates were measured during a single standardized examination visit at the NHANES Mobile Examination Center (MEC). The MEC examinations were conducted in a controlled environment using standardized protocols, with anthropometric measurements (height and weight) and blood specimen collection for serum albumin analysis performed during the same 2.5–4 h examination session. This standardized timing ensures consistency in the temporal relationship between GNRI components and reduces measurement variability that could arise from different collection timepoints.

### Definition of GNRI

The GNRI was calculated using the formula developed by Bouillanne et al.:[6]

$$\mathrm{GNRI}=\left[1.489\ast\mathrm{serum}\;\mathrm{albumin}\left(\mathrm g/\mathrm L\right)+\left[41.7\ast\left(\frac{\mathrm{actual}\;\mathrm{body}\;\mathrm{weight}}{\mathrm{ideal}\;\mathrm{body}\;\mathrm{weight}}\right)\right]\right]$$

 

Ideal body weight was calculated using the Lorentz formula:

- For men: $$\mathrm{ideal}\;\mathrm{body}\;\mathrm{weight}=\mathrm{height}\;\left(\mathrm{cm}\right)-100-\left(\frac{\mathrm{height}-150}4\right)$$ 
- For women: $$\mathrm{ideal}\;\mathrm{body}\;\mathrm{weight}=\mathrm{height}\;\left(\mathrm{cm}\right)-100-\left(\frac{\mathrm{height}-150}{2.5}\right)$$ 

In cases where the actual body weight exceeded the ideal body weight, the ratio of actual to ideal body weight was assigned a value of 1. It is important to note that GNRI is a nutrition-related risk assessment tool rather than a direct indicator of malnutrition. As originally described by Bouillanne et al., GNRI functions as a screening tool with different score ranges indicating varying levels of nutritional risk rather than definitive nutritional status [6]. For analytical purposes, GNRI values were divided into quartiles based on the distribution of GNRI scores in the study population: Q1 (65.02–100.58), Q2 (100.61–104.23), Q3 (104.24–107.20), and Q4 (107.22–122.11). This quartile approach was chosen to capture the continuous variation in GNRI scores across the large, nationally representative NHANES sample, allowing for a data-driven assessment of the dose-response relationship between GNRI and stroke prevalence. Quartile-based analyses are widely used in epidemiological studies of GNRI and stroke, as they adapt to the specific distribution of the study population and facilitate trend analyses across risk levels [22, 23]. 

### Definition of stroke

Stroke status was determined through self-reported history of physician-diagnosed stroke. Participants were asked, “Has a doctor or other health professional ever told you that you had a stroke?” with responses categorized as “yes” or “no”.

This self-reported physician-diagnosed stroke approach has been validated in multiple NHANES-based studies and demonstrates good agreement with clinical records [30–32]. However, it is important to acknowledge that this method captures only non-fatal strokes among participants who survived to the time of interview, as stroke diagnosis relies on participant self-report during the household interview or MEC examination.

### Covariates

A comprehensive set of potential confounders was considered, including demographic, socioeconomic, lifestyle, and clinical factors. Demographic variables included age (continuous), sex (male/female), and race/ethnicity. Socioeconomic factors consisted of the poverty-income ratio (PIR) and educational level. Lifestyle factors included smoking status, alcohol use, and physical activity. Clinical measurements included blood pressure, anthropometric data, and kidney function. Blood pressure was measured according to standardized procedures, with three consecutive readings averaged for analysis. Body mass index (BMI) was calculated using weight in kilograms divided by height in meters squared (kg/m²), with height measured to the nearest 0.1 cm using a stadiometer. Kidney function was assessed by the estimated glomerular filtration rate (eGFR), calculated using the Chronic Kidney Disease Epidemiology Collaboration (CKD-EPI) formula based on serum creatinine, age, sex, and race/ethnicity[33]. Comorbid conditions included hypertension, diabetes, and hyperlipidemia.

### Statistical analysis

All statistical analyses were conducted using R (version 4.4.0, http://www.R-project.org) with key packages including mgcv, rms, ggplot2, dplyr, and broom, incorporating NHANES sampling weights, stratification, and clustering variables to adjust for the complex survey design and obtain nationally representative estimates.

To address missing data in covariates, Multiple Imputation by Chained Equations (MICE) was employed, enhancing the robustness of the analysis and minimizing potential bias. The imputation model included all relevant predictors and outcome variables, applying predictive mean matching for continuous variables, logistic regression for binary variables, and multinomial regression for categorical variables. Five iterations were performed to ensure convergence.

Descriptive statistics were used to summarize baseline characteristics across GNRI quartiles. Continuous variables were expressed as survey-weighted means with 95% confidence intervals, while categorical variables were presented as survey-weighted percentages with 95% confidence intervals. Differences between groups were assessed using survey-weighted linear regression for continuous variables and survey-weighted chi-square tests for categorical variables.

To explore the relationship between GNRI and stroke, we analyzed GNRI both as a continuous variable (per one-standard-deviation increase) and as a categorical variable (quartiles). Survey-weighted logistic regression models were constructed with increasing adjustments: Model 1 (unadjusted), Model 2 (adjusted for age and sex), and Model 3 (fully adjusted for age, sex, race/ethnicity, PIR, educational level, METs/week, BMI, smoking status, drinking status, eGFR, glucose metabolism status, hypertension, and hyperlipidemia). A P for trend was calculated to assess whether there was a statistically significant trend across GNRI quartiles.

To evaluate potential nonlinear associations and ensure the robustness of our findings, we employed multiple analytical approaches as sensitivity analyses. These included generalized additive models (GAM) using the mgcv package, restricted cubic splines (RCS) using the rms package, and polynomial regression analysis [34]. Model comparisons were performed using the Akaike Information Criterion (AIC), and nonlinearity was assessed through effective degrees of freedom (edf) in GAM models and formal testing of nonlinear components in RCS models.

To assess the consistency of our findings across various subgroups, both continuous and quartile-based stratified logistic regression analyses were performed. These were stratified by key demographic and clinical factors, including age, sex, race/ethnicity, smoking status, alcohol consumption, eGFR levels, with age dichotomized at 75 years and eGFR categorized as low (< 60), moderate (60–89), and high (≥ 90) ml/min/1.73 m². For race/ethnicity, confounding adjustments in logistic regression models used a four-category variable (Non-Hispanic White, Non-Hispanic Black, Mexican American, and Others), with the ‘Others’ category comprising Other Hispanic and Other Race - Including Multi-Racial, representing the smallest classification unit in NHANES. In subgroup analyses and interaction tests, race/ethnicity was dichotomized into White (Non-Hispanic White) and Non-White (combining Non-Hispanic Black, Mexican American, and Others) to enhance statistical power and highlight the predominant White group, a common approach in NHANES-based studies [35, 36]. Effect modification was evaluated using likelihood ratio tests to assess interactions between these variables and GNRI in both continuous and categorical forms. Statistical significance was considered at a two-sided P value < 0.05.

## Results

### Study sample selection

**Fig. 1:**
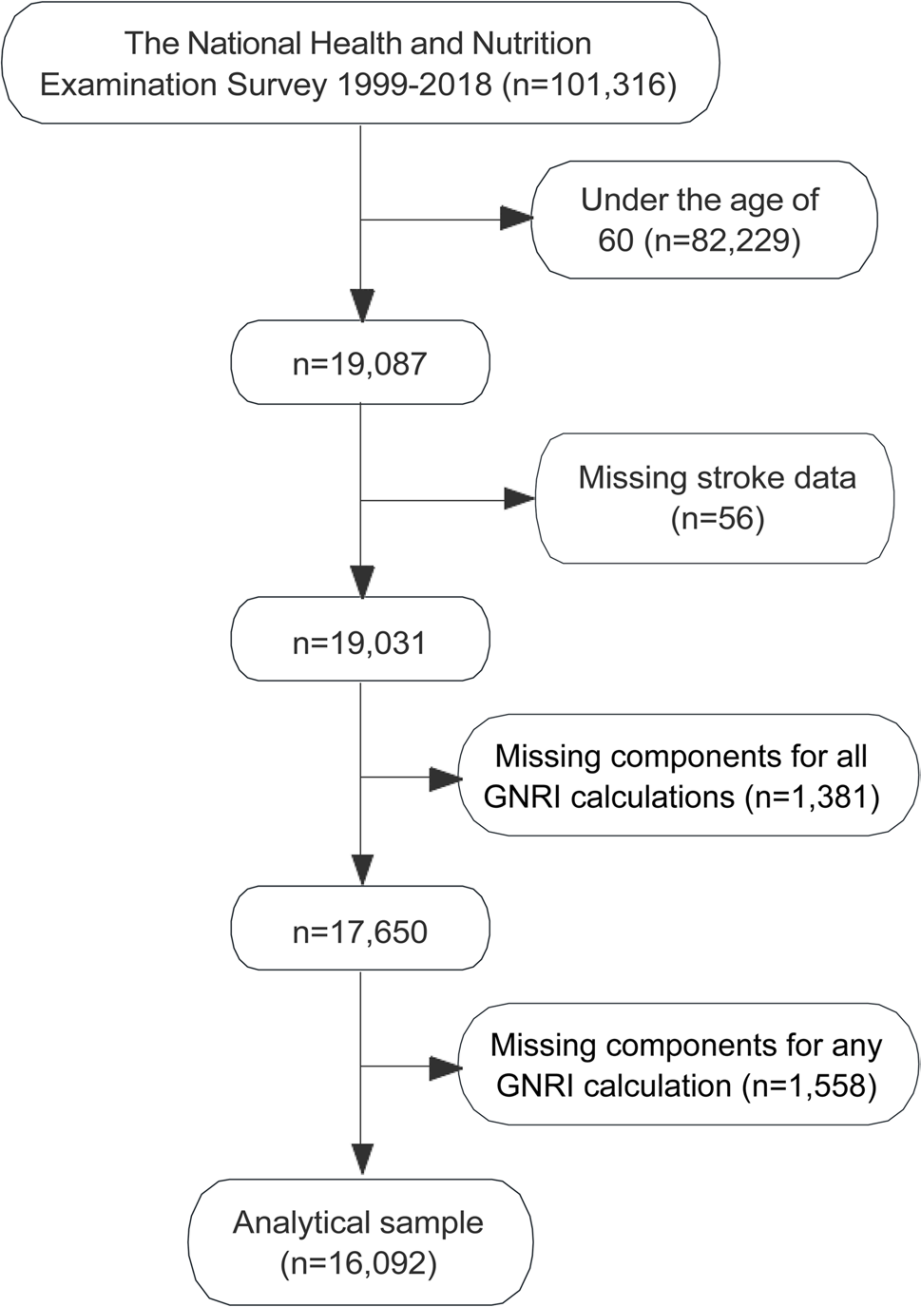
Flowchart of participant selection for the study. Abbreviations**:** GNRI, geriatric nutritional risk index

Figure 1 outlines the participant selection process for the study, utilizing data from NHANES collected between 1999 and 2018. The initial cohort included 101,316 individuals. After applying exclusion criteria, 82,229 participants under the age of 60 were removed from the analysis. Additionally, 56 participants were excluded due to missing stroke data, 1,381 participants were excluded due to missing components for all GNRI calculations, and a further 1,558 participants were excluded due to missing components for any GNRI calculation. Ultimately, the analytical sample comprised 16,092 participants, providing a robust dataset to examine the association between GNRI and stroke prevalence.

### Baseline demographic characteristics

The baseline demographic characteristics of the study participants, categorized by GNRI quartiles, are summarized in Table 1. Significant differences were observed across various variables. The average age of participants decreased progressively from the first quartile (Q1) to the fourth quartile (Q4), with the mean age in Q1 being 71.2 years and in Q4 being 69.1 years (*P* < 0.001). The gender distribution also varied significantly, with a higher percentage of males in Q2 (42.2%), Q3 (46.5%), and Q4 (53.1%) compared to Q1 (36.2%) (*P* < 0.001). Racial and ethnic composition differed, with non-Hispanic White participants increasing from 73.9% in Q1 to 82.3% in Q4 (*P* < 0.001), while non-Hispanic Black participants decreased from 12.6% in Q1 to 5.1% in Q4.

**Table 1:** Weighted baseline characteristics of study participants according to GNRI quartiles (full version with 95% confidence intervals in supplementary table S1)

Variables	GNRI quartiles				*P*-value
	Q1 (65.02–100.58) (*n* = 4023)	Q2 (100.61–104.23) (*n* = 3795)	Q3 (104.24–107.20) (*n* = 4010)	Q4 (107.22–122.11) (*n* = 4264)	
Age (years)	71.2	70.4	69.4	69.1	< 0.001
Sex (%)					< 0.001
Male	36.2	42.2	46.5	53.1	
Female	63.8	57.8	53.5	46.9	
Race/ethnicity (%)					< 0.001
Non-Hispanic White	73.9	79.1	80.8	82.3	
Non-Hispanic Black	12.6	8.6	7.0	5.1	
Mexican American	4.0	4.0	3.9	3.9	
Others	9.4	8.3	8.4	8.7	
PIR (%)					< 0.001
Low (< 1.3)	23.5	20.1	17.7	16.5	
Medium (1.3–3.5)	42.3	42.3	41.1	40.5	
High (> 3.5)	34.2	37.6	41.2	42.9	
Education level (%)					< 0.001
Less than high school	25.2	21.9	20.3	19.4	
High school graduate	27.5	25.8	24.8	26.5	
More than high school	47.4	52.3	54.9	54.1	
Smoking status (%)					< 0.001
Never	48.4	50.1	49.5	46.9	
Former	37.4	38.4	40.2	42.7	
Now	14.3	11.5	10.3	10.5	
Drinking status (%)					< 0.001
Never	19.0	17.0	15.5	13.9	
Former	27.5	22.2	22.1	22.7	
Mild	36.7	44.4	46.2	44.9	
Moderate	9.9	10.6	10.8	11.7	
Heavy	6.9	5.9	5.5	6.9	
METs/week (%)					0.027
Low (< 600)	37.0	34.4	33.5	33.5	
Moderate (600–1199)	4.0	2.7	3.6	3.6	
Vigorous (> 1199)	59.1	62.9	63.0	62.9	
SBP (mmHg)	133.9	132.4	132.9	133.8	0.026
DBP (mmHg)	66.7	67.6	68.6	69.7	< 0.001
BMI (kg/m^2^)	29.5	29.2	28.9	28.2	< 0.001
Height (cm)	163.8	165.5	166.5	167.6	< 0.001
eGFR (ml/min/1.73 m^2^)	70.0	72.3	74.1	74.6	< 0.001
Glucose metabolism state (%)					< 0.001
Normoglycemia	57.6	63.1	65.2	65.4	
Prediabetes	11.0	10.6	10.2	10.5	
Diabetes	31.5	26.3	24.5	24.1	
Hypertension (%)	69.7	67.8	66.0	67.9	0.117
Hyperlipidemia (%)	79.6	84.5	87.8	88.4	< 0.001
Stroke (%)	10.4	7.3	5.8	6.3	< 0.001
GNRI	97.2	102.1	105.0	109.2	< 0.001

*Abbreviations: GNRI* Geriatric nutritional risk index, *PIR* Poverty income ratio, *MET* Metabolic equivalent, *SBP* Systolic blood pressure, *DBP* Diastolic blood pressure, *BMI* Body mass index, *eGFR* Estimated glomerular filtration rate, *CI* Confidence interval, *HbA1c* Glycated hemoglobin, *FPG* Fasting plasma glucose, *OGTT* Oral glucose tolerance test, *LDL* Low-density lipoprotein, *HDL* High-density lipoprotein

Notes: Full notes in Supplementary Table S1

Socioeconomic status, as measured by the PIR, indicated that low-income participants were more common in Q1 (23.5%) than in Q2 (20.1%) or Q3 (17.7%) (*P* < 0.001). Educational attainment also varied significantly, with the proportion of participants with less than a high school education declining from 25.2% in Q1 to 19.4% in Q4 (*P* < 0.001).

Lifestyle factors, including smoking and alcohol consumption, showed notable differences across GNRI quartiles. The percentage of current smokers decreased from 14.3% in Q1 to 10.5% in Q4 (*P* < 0.001). Alcohol consumption patterns also varied, with the percentage of participants who had never consumed alcohol decreasing from 19.0% in Q1 to 13.9% in Q4 (*P* < 0.001).

Clinical characteristics such as blood pressure, BMI, and kidney function exhibited significant differences across GNRI quartiles. Systolic blood pressure (SBP) was highest in Q1 at 133.9 mmHg and lowest in Q2 at 132.4 mmHg(*P* = 0.026). BMI decreased from 29.5 kg/m² in Q1 to 28.2 kg/m² in Q4 (*P* < 0.001). The eGFR showed an improvement from Q1 to Q4, with 70.0 ml/min/1.73 m² in Q1 and 74.6 ml/min/1.73 m² in Q4 (*P* < 0.001). Additionally, the prevalence of diabetes decreased significantly across GNRI quartiles from 31.5% in Q1 to 24.1% in Q4 (*P* < 0.001), while hypertension showed no significant difference across quartiles (69.7% in Q1 to 67.9% in Q4, *P* = 0.117).

### Association between GNRI and stroke

The analysis of the association between GNRI and stroke in the elderly population, as shown in Table 2, demonstrates a significant inverse relationship. For each one-standard-deviation increase in GNRI, the odds of stroke were lower, with odds ratios (ORs) of 0.81 (95% CI: 0.76–0.85) in Model 1, 0.82 (95% CI: 0.78–0.87) in Model 2, and 0.88 (95% CI: 0.83–0.93) in Model 3.

**Table 2:** Weighted analysis of the association between GNRI and stroke among elderly population

GNRI	OR (95% CI)		
	Model 1	Model 2	Model 3
Continuous (per one SD)	0.81 (0.76, 0.85)	0.82 (0.78, 0.87)	0.88 (0.83, 0.93)
Quartiles			
Q1 (65.02–100.58)	Reference	Reference	Reference
Q2(100.61–104.23)	0.71 (0.61, 0.83)	0.73 (0.62, 0.85)	0.81 (0.69, 0.95)
Q3(104.24–107.20)	0.62 (0.53, 0.73)	0.65 (0.55, 0.76)	0.74 (0.63, 0.87)
Q4(107.22–122.11)	0.62 (0.53, 0.72)	0.66 (0.57, 0.77)	0.77 (0.65, 0.91)
P for trend	< 0.05	< 0.05	< 0.05

*Abbreviations: GNRI* Geriatric nutritional risk index, *OR* Odds ratio, *CI* Confidence interval, *SD* Standard deviation, *PIR* Poverty income ratio, *BMI* Body mass index, *MET* Metabolic equivalent, *eGFR* Estimated glomerular filtration rate

Model 1: Non-adjusted

Model 2: Adjusted for age and sex

Model 3: Adjusted for age, sex, race/ethnicity, PIR, education level, BMI, smoking status, drinking status, glucose metabolism state, hypertension, hyperlipidemia, METs/week, and eGFR

Regarding GNRI quartiles, participants in the second quartile (Q2, 100.61–104.23) had ORs of 0.71 (95% CI: 0.61–0.83) in Model 1, 0.73 (95% CI: 0.62–0.85) in Model 2, and 0.81 (95% CI: 0.69–0.95) in Model 3, compared to those in the first quartile (Q1, 65.02–100.58). In the third quartile (Q3, 104.24–107.20), the ORs were 0.62 (95% CI: 0.53–0.73) in Model 1, 0.65 (95% CI: 0.55–0.76) in Model 2, and 0.74 (95% CI: 0.63–0.87) in Model 3. Participants in the fourth quartile (Q4, 107.22–122.11) had ORs of 0.62 (95% CI: 0.53–0.72) in Model 1, 0.66 (95% CI: 0.57–0.77) in Model 2, and 0.77 (95% CI: 0.65–0.91) in Model 3. The trend across GNRI quartiles was statistically significant (P for trend < 0.05).

**Fig. 2:**
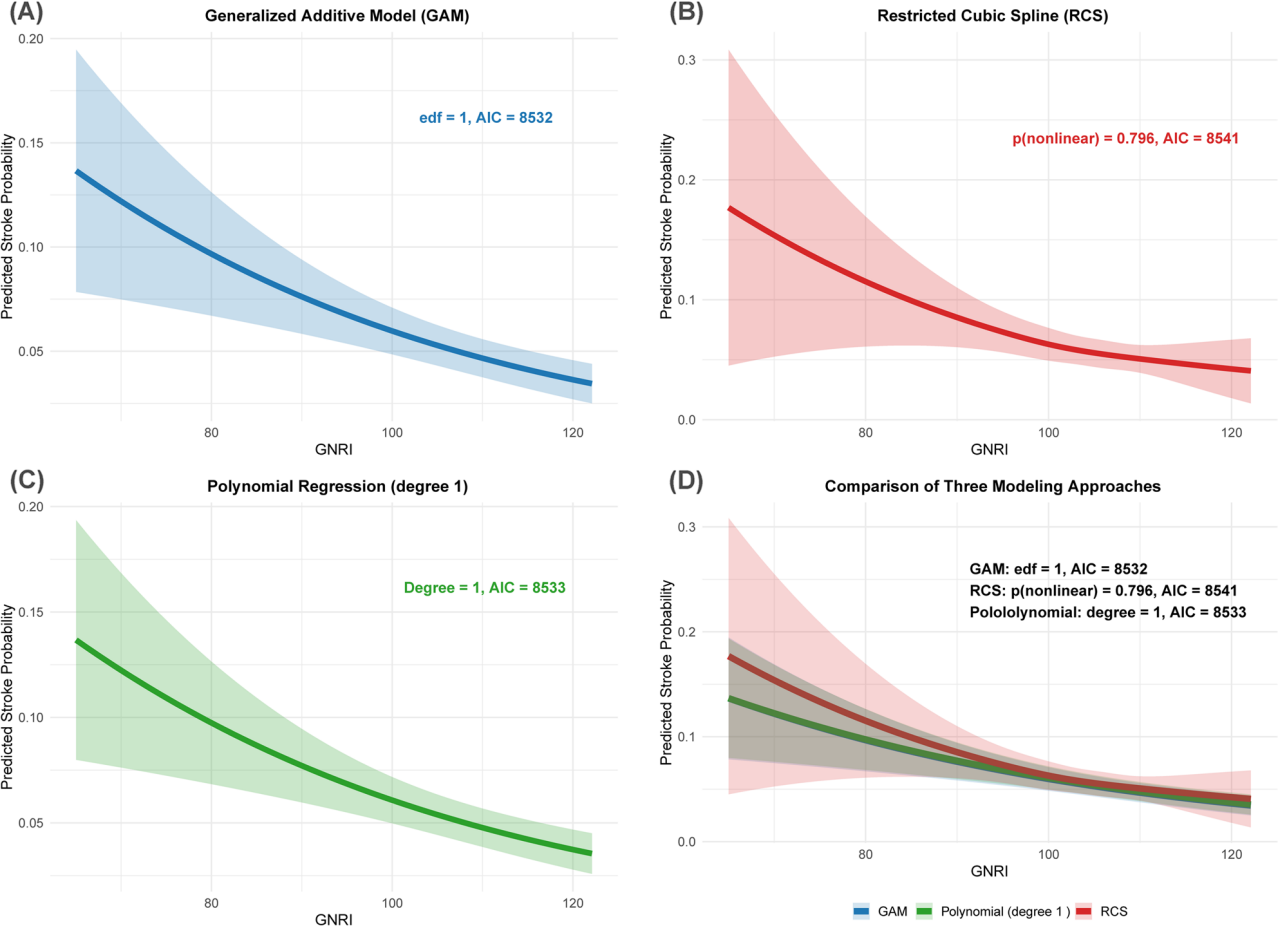
Comparison of three modeling approaches for the association between GNRI and stroke prevalence Notes: This figure depicts the relationship between GNRI and predicted stroke probability using three different modeling approaches: (**A**) GAM, (**B**) RCS, (**C**) polynomial regression (degree 1), and (**D**) all three models superimposed, with adjustments for confounders including age, sex, race/ethnicity, PIR, education level, BMI, smoking status, drinking status, glucose metabolism state, hypertension, hyperlipidemia, METs/week, and eGFR. Colors indicate modeling method: deep blue for GAM, deep red for RCS, and deep green for polynomial regression. Shaded areas show 95% CIs for each model. Abbreviations: GNRI, geriatric nutritional risk index; GAM, generalized additive model; RCS, restricted cubic splines; AIC, akaike information criterion; edf, effective degrees of freedom; PIR, poverty income ratio; MET, metabolic equivalent; BMI, body mass index; eGFR, estimated glomerular filtration rate; CI, confidence interval

To assess potential nonlinear associations, we employed GAM, RCS, and polynomial regression analysis, as shown in Fig. 2. Model comparison revealed that GAM (AIC = 8532), RCS (AIC = 8541), and polynomial regression (AIC = 8533) yielded similar results, with RCS analysis indicating no significant nonlinearity (*P* = 0.796). All three analytical approaches consistently demonstrated a linear inverse association between higher GNRI values and lower stroke probability, supporting the robustness of the linear relationship observed in the logistic regression analysis.

**Fig. 3:**
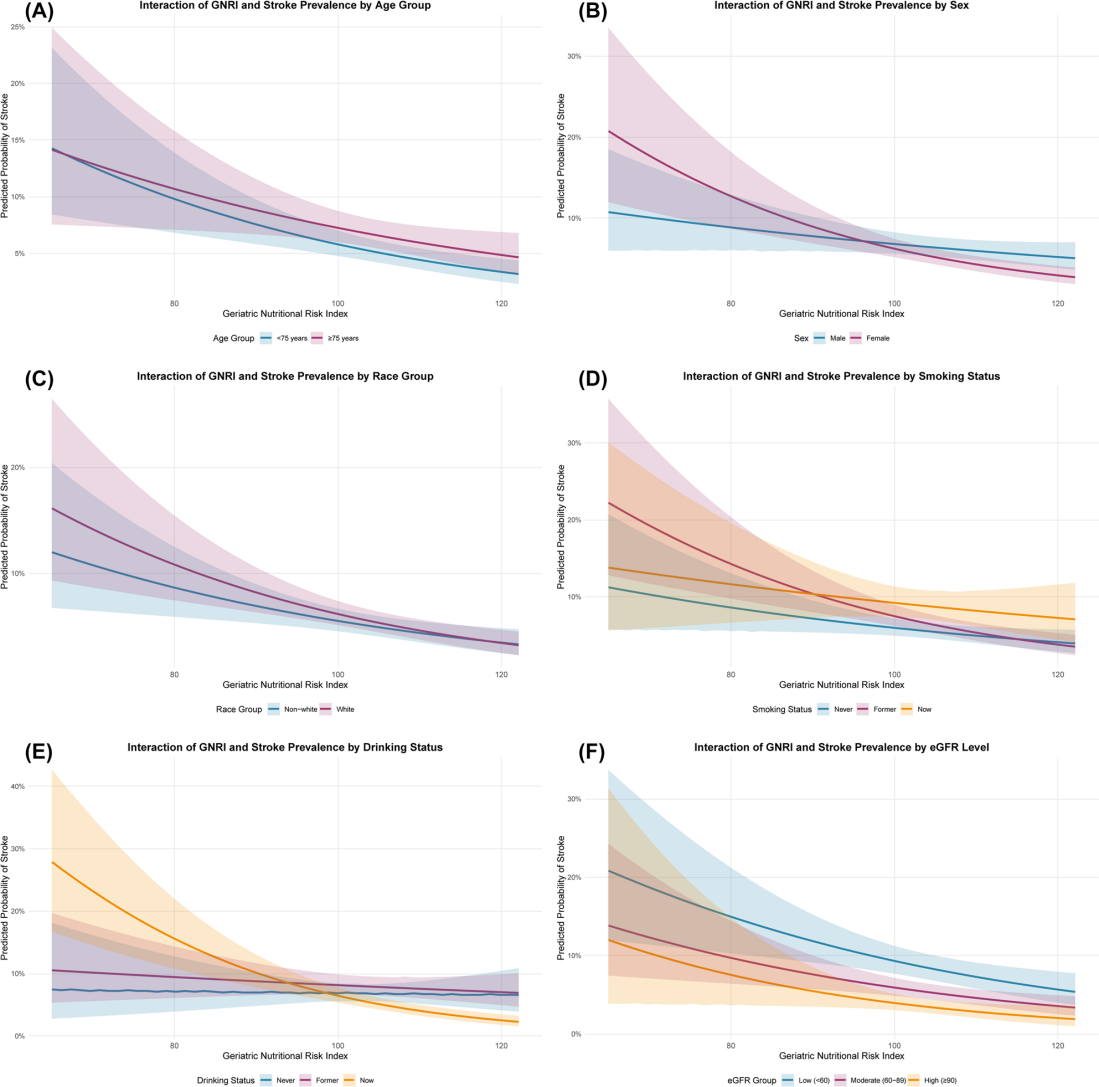
Interaction analysis of GNRI and stroke probability across multiple subgroups Notes: This figure depicts logistic regression models assessing the relationship between GNRI and stroke probability across subgroups defined by (**A**) age, (**B**) sex, (**C**) race/ethnicity, (**D**) smoking status, (**E**) drinking status, and (**F**) eGFR, with adjustments for confounders including age, sex, race/ethnicity, PIR, education level, BMI, smoking status, drinking status, glucose metabolism state, hypertension, hyperlipidemia, METs/week, and eGFR. Colors indicate subgroups within each variable, with distinct hues for each level. Shaded areas show 95% CIs for predicted probabilities. Abbreviations: GNRI, geriatric nutritional risk index; PIR, poverty income ratio; MET, metabolic equivalent; BMI, body mass index; eGFR, estimated glomerular filtration rate; CI, confidence interval

Subgroup analyses explored the consistency of the GNRI-stroke association across various demographic and clinical characteristics, as presented in Table 3; Fig. 3. The association remained significant across most subgroups, with notable effect modification observed in specific populations. Sex showed a significant interaction (*P* = 0.03), with a stronger inverse association in females (OR: 0.96, 95% CI: 0.95–0.98) compared to males (OR: 0.99, 95% CI: 0.97–1.00). Drinking status also demonstrated significant effect modification (*P* = 0.001), with the strongest inverse association observed among current drinkers (OR: 0.96, 95% CI: 0.94–0.97), while no significant association was found among never drinkers (OR: 1.00, 95% CI: 0.97–1.02). Race/ethnicity showed no significant interaction (*P* = 0.56), with both Non-White (OR: 0.97, 95% CI: 0.96–0.99) and White (OR: 0.97, 95% CI: 0.96–0.99) participants demonstrating similar inverse associations for continuous GNRI, using a dichotomized White/Non-White classification as detailed under Statistical analysis. Table 1 shows that Non-Hispanic White participants predominated (73.92–82.28%) across GNRI quartiles, while the ‘Others’ category (8.28–9.43%), comprising Other Hispanic and Other Race - Including Multi-Racial, was larger than Mexican Americans (3.75–4.04%)[29]. Other subgroups, including age, smoking status, and eGFR levels, showed consistent inverse associations without significant interactions. Figure 3 visually depicts these subgroup-specific relationships, demonstrating that while the overall inverse trend is maintained across subgroups, the magnitude of association varies by specific demographic and lifestyle characteristics.

**Table 3:** Subgroup analysis of association between GNRI and stroke

Subgroup variable	Subgroup category	OR (95% CI)	Level-specific interaction *P*-value	Overall interaction *P*-value
Age (years)				0.54
	< 75	0.97 (0.96–0.99)	-	
	≥ 75	0.98 (0.96–1.00)	0.54	
Sex				0.03
	Male	0.99 (0.97–1.00)	-	
	Female	0.96 (0.95–0.98)	0.03	
Race/ethnicity				0.56
	Non-white	0.97 (0.96–0.99)	-	
	White	0.97 (0.96–0.99)	0.57	
Smoking status				0.24
	Never	0.98 (0.96–1.00)	-	
	Former	0.96 (0.95–0.98)	0.19	
	Current	0.99 (0.96–1.02)	0.68	
Drinking status				0.001
	Never	1.00 (0.97–1.02)	-	
	Former	0.99 (0.97–1.01)	0.72	
	Current	0.96 (0.94–0.97)	0.003	
eGFR (ml/min/1.73 m^2^)				0.91
	Low (< 60)	0.97 (0.96–0.99)	-	
	Moderate (60–89)	0.97 (0.96–0.99)	0.99	
	High (≥ 90)	0.97 (0.94–1.00)	0.68	

*Abbreviations: GNRI* Geriatric nutritional risk index, *OR* Odds ratio, *CI* Confidence interval, *eGFR* Estimated glomerular filtration rate, *PIR* Poverty income ratio, *MET* Metabolic equivalent of task, *BMI* body mass index

Notes: Detailed model adjustments, interaction test explanations, and abbreviations are provided in Supplementary Text 1

### Sensitivity analysis

To further validate our findings, we conducted comprehensive sensitivity analyses examining the association between GNRI quartiles and stroke across various subgroups (Table S2 and Figure S1). The quartile-based subgroup analysis revealed several significant interactions that complement our continuous GNRI findings. Age demonstrated significant effect modification (*P* = 0.02), with consistent inverse associations in participants aged < 75 years across Q3 (OR: 0.72, 95% CI: 0.57–0.90) and Q4 (OR: 0.71, 95% CI: 0.55–0.90), while participants aged ≥ 75 years showed a different pattern with the strongest inverse associations in Q2 and Q4. Race/ethnicity also showed significant interaction (*P*= 0.03), with non-White participants demonstrating consistent inverse associations across all quartiles, particularly in Q3 (OR: 0.62, 95% CI: 0.48–0.80), using a dichotomized White/Non-White classification as detailed under Statistical analysis [29]. The most pronounced interaction was observed for drinking status (*P* = 0.008), where current drinkers exhibited the strongest inverse associations across all quartiles (Q2-Q4 ORs: 0.67, 0.60, 0.54, respectively), while never drinkers showed no significant associations. Sex, smoking status, and eGFR levels showed no significant interactions, confirming the consistency of the GNRI-stroke association across these characteristics. These sensitivity analyses reinforce our primary findings while revealing important effect modifications by age, race/ethnicity, and alcohol consumption patterns.

Given the significant age-related interaction observed above, we conducted more detailed age-stratified analyses using 70 years as the cutoff point to better understand the consistency of our findings within the elderly population (Table S3). The inverse association between GNRI and stroke prevalence remained consistent in both age groups. In participants aged 60–69 years, each one-standard-deviation increase in GNRI was associated with a 13% lower stroke odds (OR: 0.87, 95% CI: 0.79–0.95, *P* = 0.003), while in participants aged ≥ 70 years, the association was similarly inverse, with 11% lower stroke odds (OR: 0.89, 95% CI: 0.83–0.96, *P* = 0.002). These findings demonstrate that the GNRI-stroke association is robust across different age strata within the elderly population.

Building on these age-related findings, we also examined whether our results would remain consistent when applying the traditional definition of elderly population. We conducted an additional analysis restricting our sample to participants aged ≥ 65 years, following the conventional elderly population criteria and the original GNRI validation standards (Table S4). This sensitivity analysis included 11,622 participants aged ≥ 65 years and confirmed the inverse association between GNRI and stroke prevalence. Each one-standard-deviation increase in GNRI was associated with 10% lower stroke odds (OR: 0.90, 95% CI: 0.85–0.96, *P* = 0.001). Similarly, the quartile analysis showed consistent inverse associations across Q2 (OR: 0.81, 95% CI: 0.68–0.97), Q3 (OR: 0.76, 95% CI: 0.63–0.91), and Q4 (OR: 0.82, 95% CI: 0.69–0.99) compared to the lowest quartile. These findings confirm that our results are robust when restricted to the traditionally defined elderly population.

Finally, we examined the potential methodological concern regarding BMI inclusion in our fully adjusted model, given that BMI components (weight and height) overlap with GNRI calculation. We conducted a sensitivity analysis excluding BMI from Model 3 to evaluate whether this covariate adjustment might introduce bias (Table S5). The comparison revealed highly consistent results between models with and without BMI adjustment, with OR estimates differing only slightly when reported to three decimal places (e.g., continuous GNRI OR: 0.880 with BMI vs. 0.882 without BMI). These findings indicate negligible overadjustment bias and support the appropriateness of including BMI as a confounder in our primary analysis.

## Discussion

In this extensive analysis of 16,092 elderly participants from NHANES (1999–2018), we identified a significant linear inverse association between higher GNRI values and lower stroke prevalence. Our investigation employed multiple analytical approaches, including GAM, RCS, and polynomial regression, which consistently demonstrated a linear inverse association rather than a nonlinear association. It is important to note that GNRI serves as a nutritional risk assessment tool rather than a direct diagnostic indicator of malnutrition, as designed by its original developers to classify patients according to risk of nutrition-related mortality. Higher GNRI values were associated with lower odds of prevalent stroke after full adjustment. Quartile analysis showed progressively lower odds in higher GNRI quartiles compared to the lowest quartile. Importantly, our stratified analyses revealed significant effect modification by sex and drinking status in continuous analyses, with quartile-based sensitivity analyses additionally identifying age and race/ethnicity interactions, underscoring the heterogeneity of the inverse association across different demographic and lifestyle subgroups while maintaining an overall inverse trend between nutritional risk levels and stroke prevalence.

Our results are in line with, and extend, previous research on the relationship between GNRI and cardiovascular outcomes. Several studies have shown that lower GNRI values are associated with adverse health outcomes in different populations. Cai et al. reported that low GNRI was linked to an increased risk of incident stroke in elderly hypertensive patients,[22] while Tsuneyoshi et al. found low GNRI to be an independent risk factor for both brain infarction and hemorrhage in hemodialysis patients [23]. Similarly, Dai et al. observed that lower GNRI scores were associated with a higher prevalence of stroke-related pneumonia [37]. Additionally, meta-analyses have indicated that lower GNRI values are linked to an increased risk of all-cause mortality and major cardiovascular events in heart failure patients [16]. Our study contributes to this body of work by demonstrating a consistent linear inverse association between higher GNRI values and lower stroke prevalence in a large, nationally representative elderly population, using multiple analytical approaches to ensure the robustness of this linear relationship.

The observed linear inverse association between higher GNRI values and lower stroke prevalence can be explained by several interconnected biological mechanisms. The GNRI incorporates both serum albumin levels and body weight relative to ideal weight, providing a comprehensive assessment of nutrition-related risk that may influence cerebrovascular health [6]. Higher GNRI values indicate lower nutritional risk, which is associated with lower prevalence of vascular complications and greater physiological resilience. Individuals with lower nutritional risk, as reflected by higher GNRI scores, may exhibit enhanced anti-inflammatory and antioxidant functions, which could be linked to lower odds of cerebrovascular damage [38]. 

Lower malnutrition risk is associated with improved immune function, enhanced vascular integrity, and better regulation of inflammatory processes, all of which may be linked to lower stroke prevalence [39, 40]. Additionally, reduced nutritional risk is associated with optimal brain plasticity and neuroprotein synthesis, which may help maintain cerebrovascular health and is linked to lower stroke prevalence [21]. Besides the GNRI-stroke relationship, our analysis also identified that the mean eGFR was below 75 ml/min/1.73 m² across all GNRI quartiles (ranging from 69.97 in Q1 to 74.56 in Q4), consistent with age-related kidney function decline commonly observed in elderly populations [41, 42]. The observed trend of increasing eGFR with higher GNRI quartiles suggests that lower nutritional risk may be associated with better kidney function, potentially due to reduced systemic inflammation and improved metabolic health [43, 44]. This aligns with studies linking poor nutritional status to chronic kidney disease progression and cardiovascular risk, although the direct relationship between eGFR and stroke prevalence in this context warrants further investigation. The consistent linear inverse association observed across multiple analytical approaches suggests that lower nutritional risk, as assessed by GNRI, is associated with progressively lower stroke prevalence across the GNRI range. While the inverse association was evident across higher GNRI quartiles, the relationship demonstrates that each increment in GNRI corresponds to lower stroke prevalence, supporting the clinical utility of GNRI as a nutritional risk stratification tool in elderly populations. This finding aligns with observations of inverse associations between various nutritional factors and stroke risk, such as those seen with antioxidants and dietary inflammatory indices, suggesting shared underlying mechanisms between nutrition and cerebrovascular health [45–48]. 

Our subgroup analyses revealed several important insights regarding the relationship between GNRI and stroke risk across different populations. The linear inverse association between higher GNRI values and lower stroke prevalence showed meaningful heterogeneity across specific demographic and lifestyle subgroups, with notable effect modifications observed in key populations. Sex demonstrated significant interaction effects (*P* = 0.03), with a stronger inverse association in females compared to males, which aligns with previous findings that men experience higher stroke incidence and may face different risk factor profiles [49, 50]. Drinking status also showed significant effect modification (*P* = 0.001), with the strongest inverse association observed among current drinkers, while no significant association was found among never drinkers, potentially reflecting complex interactions between alcohol consumption patterns and nutritional risk factors [51, 52]. Additionally, quartile-based sensitivity analyses revealed significant effect modifications by age (*P* = 0.02) and race/ethnicity (*P* = 0.03), while smoking status and eGFR levels showed consistent inverse associations across subgroups without significant interactions. These findings highlight the importance of considering individual characteristics when interpreting GNRI values and designing nutritional risk screening or intervention strategies [53]. 

The clinical implications of our findings are substantial and multifaceted. First, they underscore the critical role of nutritional risk assessment in stroke risk stratification assessment and highlight the importance of utilizing simple and practical tools, such as the GNRI, for the early identification of high-risk elderly individuals at potentially higher risk. The demonstration of a linear inverse association across the entire GNRI range provides clinicians with evidence that systematic nutritional screening can effectively stratify stroke prevalence in elderly populations. Second, given that GNRI incorporates both serum albumin levels and body weight relative to ideal weight, our findings suggest that clinical interventions targeting these specific components may be of interest in geriatric care. The progressively lower odds observed across GNRI quartiles indicates that strategies aimed at optimizing protein nutritional status through albumin improvement and maintaining appropriate weight-to-height ratios could serve as observable factors associated with lower stroke prevalence in elderly patients. This offers direct guidance for policymakers to incorporate routine nutritional risk screening into healthy aging and cerebrovascular health programs. Third, the significant effect modifications observed by sex, alcohol consumption status, age, and race/ethnicity highlight the importance of personalized nutritional risk assessment when interpreting GNRI values and designing targeted screening approaches for different patient populations.

Before interpreting these findings for clinical practice, it is worth noting an important aspect of GNRI interpretation in older adults. We acknowledge that GNRI does not directly assess muscle mass or body composition. Consequently, phenotypes such as sarcopenic obesity—a condition increasingly recognized in older adults—may complicate the interpretation of GNRI values. Curcio et al. first proposed the concept of a “reverse metabolic syndrome” or “catabolic syndrome” in the elderly, characterized by loss of muscle mass despite preserved or increased fat mass, resulting in lower GNRI scores even when BMI is normal or high [54]. This concept has been further supported in recent studies, which demonstrate that low GNRI independently predicts sarcopenia and related outcomes in older adults with chronic conditions, including diabetes and stroke [17, 55]. This phenotype could partly explain the observed inverse association between higher GNRI and lower stroke prevalence in our study, as it highlights the shift from anabolic to catabolic patterns with aging and underscores the need to consider muscle quality alongside traditional nutritional markers.

However, several limitations must be acknowledged. First, the cross-sectional design precludes causal inference and raises the major concern of reverse causation. Stroke survivors may experience dysphagia, depression, reduced appetite, metabolic disturbances, or decreased mobility—all of which can lead to weight loss and hypoalbuminemia, thereby lowering GNRI values after the stroke event rather than before it. Although two prospective studies have suggested that low baseline GNRI predicts incident stroke[22, 23], the possibility that post-stroke nutritional deterioration contributes to the observed association in the present cross-sectional analysis cannot be excluded. Second, although we adjusted for a range of potential confounders, residual confounding by unmeasured factors—such as atrial fibrillation, antiplatelet/anticoagulant medication use, and systemic inflammatory markers (e.g., C-reactive protein, available only in selected cycles)—remains possible. Furthermore, NHANES does not differentiate ischemic from hemorrhagic stroke, which have distinct pathophysiological mechanisms and risk factor profiles; the observed association may therefore differ by stroke subtype. Additionally, the reliance on self-reported physician-diagnosed stroke history introduces the potential for recall bias and misclassification, and can only capture non-fatal events among survivors. Although the self-reported stroke definition has been validated in NHANES-based studies and shows good agreement with clinical records[30–32], this bias could affect the accuracy of stroke prevalence estimates. Finally, the reliance on secondary data from NHANES limits the ability to assess detailed clinical variables and nutritional factors that might further elucidate the mechanisms underlying the observed association. Despite these limitations, our study contributes important evidence regarding the cross-sectional association between nutritional risk and stroke prevalence, with implications for public health and clinical practice in aging populations.

Looking ahead, several important directions for future research emerge from our findings. Prospective cohort studies are needed to clarify the temporal relationship between GNRI and incident stroke and to examine whether the linear dose-response association observed in this cross-sectional analysis is confirmed in longitudinal settings. Investigating more comprehensive nutritional assessment tools that include biomarkers of inflammation and micronutrient status could improve the accuracy of nutritional evaluation. Future studies should also explore the relationship between GNRI and specific stroke subtypes to determine whether the observed associations differ by stroke type. Additionally, mechanistic studies are warranted to understand the biological pathways underlying the significant effect modifications observed by sex, alcohol consumption status, age, and race/ethnicity, which could help interpret GNRI values in different population subgroups. Furthermore, future research should also explore the reverse relationship, investigating whether stroke occurrence influences subsequent nutritional risk status as assessed by GNRI, which could inform post-stroke nutritional management strategies and provide insights into the potential bidirectional relationship between nutritional status and cerebrovascular health.

## Conclusion

In conclusion, this large, nationally representative cross-sectional study of US older adults demonstrated a significant linear inverse association between higher GNRI values and lower stroke prevalence, with progressively lower odds observed across higher GNRI quartiles. The association was particularly pronounced in females and current drinkers. These findings support the potential utility of GNRI as a simple nutritional risk screening tool in geriatric populations and highlight the importance of considering individual characteristics (sex, alcohol consumption, age, and race/ethnicity) when interpreting GNRI values in clinical practice. However, GNRI reflects nutritional risk rather than diagnosed malnutrition, and due to the cross-sectional design, causality cannot be inferred, and reverse causation remains possible. Prospective longitudinal studies are required to determine whether improvements in GNRI components are associated with reduced incident stroke risk.

## Supplementary Information

Supplementary Material 1

## Data Availability

Publicly available datasets were analyzed in this study. This data can be found here: NHANES website (https://www.cdc.gov/nchs/nhanes/index.htm).

## Abbreviations

GNRI: geriatric nutritional risk index
PIR: poverty income ratio
MET: metabolic equivalent
SBP: systolic blood pressure
DBP: diastolic blood pressure
BMI: body mass index
eGFR: estimated glomerular filtration rate
CI: confidence interval
HbA1c: glycated hemoglobin
FPG: fasting plasma glucose
OGTT: oral glucose tolerance test
LDL: low-density lipoprotein
HDL: high-density lipoprotein
