# Supplementary material for: Association between geriatric nutritional risk index and stroke prevalence in elderly adults: a cross-sectional analysis of NHANES 1999–2018
Source: BMC Geriatr. 2026 Mar 25;26:628. doi: 10.1186/s12877-025-06959-6 (PMC13141278; doi:10.1186/s12877-025-06959-6)
Supplement: Supplementary file 1 — Supplementary Material 1 [file 12877_2025_6959_MOESM1_ESM.docx]

**Table S1. Detailed weighted baseline characteristics of study participants according to GNRI quartiles, including 95% confidence intervals and complete notes**

| **Variables** | **GNRI quartiles^a^** | | | | **P-value** |
| --- | --- | --- | --- | --- | --- |
|  | **Q1 (65.02-100.58) (n=4023)** | **Q2 (100.61-104.23) (n=3795)** | **Q3 (104.24-107.20) (n=4010)** | **Q4 (107.22-122.11) (n=4264)** |  |
| **Age (years)** | 71.17 (70.82, 71.51) | 70.42 (70.05, 70.78) | 69.44 (69.14, 69.74) | 69.06 (68.75, 69.36) | <0.001 |
| **Sex (%)** |  |  |  |  | <0.001 |
| Male | 36.17 (34.25, 38.13) | 42.22 (40.22, 44.24) | 46.49 (44.67, 48.31) | 53.13 (51.29, 54.96) |  |
| Female | 63.83 (61.87, 65.75) | 57.78 (55.76, 59.78) | 53.51 (51.69, 55.33) | 46.87 (45.04, 48.71) |  |
| **Race/ethnicity (%)** | | | | | <0.001 |
| Non-Hispanic White | 73.92 (71.56, 76.15) | 79.14 (76.81, 81.29) | 80.80 (78.49, 82.91) | 82.28 (80.32, 84.09) |  |
| Non-Hispanic Black | 12.61 (11.08, 14.32) | 8.59 (7.31, 10.06) | 7.04 (6.10, 8.12) | 5.10 (4.37, 5.95) |  |
| Mexican American | 4.04 (3.25, 5.00) | 4.00 (3.20, 4.98) | 3.75 (2.98, 4.70) | 3.93 (3.12, 4.94) |  |
| Others | 9.43 (8.07, 10.99) | 8.28 (7.20, 9.50) | 8.41 (7.15, 9.87) | 8.68 (7.49, 10.05) |  |
| **PIR (%)** | | | | | <0.001 |
| Low (<1.3) | 23.46 (21.67, 25.35) | 20.11 (18.30, 22.06) | 17.67 (16.07, 19.39) | 16.54 (14.79, 18.46) |  |
| Medium (1.3-3.5) | 42.31 (39.82, 44.85) | 42.27 (40.15, 44.43) | 41.13 (38.53, 43.79) | 40.53 (38.20, 42.90) |  |
| High (>3.5) | 34.22 (31.42, 37.15) | 37.61 (34.99, 40.31) | 41.20 (38.22, 44.24) | 42.93 (40.26, 45.63) |  |
| **Education level (%)** | | | | | <0.001 |
| Less than high school | 25.20 (23.38, 27.11) | 21.85 (19.79, 24.05) | 20.28 (18.56, 22.12) | 19.38 (17.57, 21.33) |  |
| High school graduate | 27.45 (25.44, 29.55) | 25.81 (23.80, 27.93) | 24.83 (22.84, 26.93) | 26.53 (24.54, 28.62) |  |
| More than high school | 47.35 (45.02, 49.70) | 52.34 (49.63, 55.04) | 54.89 (52.14, 57.61) | 54.09 (51.47, 56.69) |  |
| **Smoking status (%)^b^** | | | | | <0.001 |
| Never | 48.35 (46.39, 50.31) | 50.09 (47.88, 52.29) | 49.50 (47.42, 51.58) | 46.87 (44.56, 49.19) |  |
| Former | 37.36 (35.51, 39.24) | 38.38 (36.32, 40.48) | 40.17 (38.11, 42.27) | 42.68 (40.49, 44.90) |  |
| Now | 14.29 (12.70, 16.05) | 11.53 (10.24, 12.97) | 10.33 (9.04, 11.79) | 10.45 (9.14, 11.91) |  |
| **Drinking status (%)^c^** | | | | | <0.001 |
| Never | 19.04 (17.36, 20.84) | 16.97 (15.48, 18.58) | 15.49 (13.84, 17.29) | 13.87 (12.25, 15.67) |  |
| Former | 27.45 (25.63, 29.34) | 22.18 (20.48, 23.99) | 22.09 (20.26, 24.04) | 22.69 (20.64, 24.89) |  |
| Mild | 36.65 (34.64, 38.71) | 44.42 (42.31, 46.55) | 46.16 (43.72, 48.63) | 44.89 (42.54, 47.26) |  |
| Moderate | 9.94 (8.40, 11.73) | 10.57 (9.34, 11.94) | 10.77 (9.32, 12.41) | 11.69 (10.28, 13.27) |  |
| Heavy | 6.92 (5.89, 8.11) | 5.86 (4.82, 7.09) | 5.49 (4.57, 6.59) | 6.85 (5.77, 8.12) |  |
| **METs/week (%)** | | | | | 0.027 |
| Low (<600) | 36.96 (35.13, 38.82) | 34.42 (32.23, 36.67) | 33.45 (31.35, 35.62) | 33.52 (31.52, 35.58) |  |
| Moderate (600-1199) | 3.97 (3.09, 5.09) | 2.65 (2.05, 3.42) | 3.60 (2.88, 4.49) | 3.58 (2.91, 4.40) |  |
| Vigorous (>1199) | 59.07 (57.24, 60.88) | 62.93 (60.56, 65.24) | 62.95 (60.64, 65.20) | 62.90 (60.82, 64.94) |  |
| **SBP (mmHg)** | 133.92 (132.98, 134.86) | 132.43 (131.52, 133.34) | 132.88 (132.00, 133.76) | 133.75 (132.82, 134.69) | 0.026 |
| **DBP (mmHg)** | 66.68 (66.07, 67.29) | 67.56 (66.91, 68.21) | 68.60 (68.05, 69.16) | 69.68 (69.12, 70.23) | <0.001 |
| **BMI (kg/m^2^)** | 29.45 (29.12, 29.79) | 29.24 (28.93, 29.55) | 28.92 (28.68, 29.15) | 28.23 (28.04, 28.42) | <0.001 |
| **Height (cm)** | 163.77 (163.34, 164.21) | 165.52 (165.10, 165.94) | 166.49 (166.07, 166.91) | 167.59 (167.16, 168.02) | <0.001 |
| **eGFR (ml/min/1.73 m^2^)** | 69.97 (69.10, 70.83) | 72.26 (71.46, 73.05) | 74.11 (73.30, 74.92) | 74.56 (73.89, 75.22) | <0.001 |
| **Glucose metabolism state (%)^d^** | | | | | <0.001 |
| Normoglycemia | 57.58 (55.17, 59.95) | 63.14 (60.87, 65.36) | 65.23 (63.04, 67.36) | 65.42 (63.46, 67.33) |  |
| Prediabetes | 10.97 (9.47, 12.68) | 10.57 (9.17, 12.16) | 10.24 (8.93, 11.72) | 10.48 (9.03, 12.14) |  |
| Diabetes | 31.45 (29.29, 33.69) | 26.28 (24.39, 28.27) | 24.52 (22.68, 26.46) | 24.10 (22.40, 25.88) |  |
| **Hypertension (%)^e^** | 69.67 (67.54, 71.71) | 67.75 (65.34, 70.07) | 66.03 (63.81, 68.19) | 67.92 (65.97, 69.82) | 0.117 |
| **Hyperlipidemia (%)^f^** | 79.57 (77.80, 81.24) | 84.48 (82.76, 86.05) | 87.76 (85.90, 89.40) | 88.36 (87.12, 89.49) | <0.001 |
| **Stroke (%)** | 10.39 (9.20, 11.71) | 7.27 (6.27, 8.41) | 5.84 (4.90, 6.94) | 6.30 (5.49, 7.22) | <0.001 |
| **GNRI** | 97.22 (97.11, 97.34) | 102.13 (102.09, 102.16) | 105.00 (104.96, 105.03) | 109.19 (109.09, 109.28) | <0.001 |

**Notes:** ^a^ Continuous variables are reported as survey-weighted mean values (with 95% CIs), with P-values derived using survey-weighted linear regression analysis (svyglm), while categorical variables are presented as survey-weighted percentage distributions (with 95% CIs) and P-values calculated through survey-weighted Chi-square testing (svytable).

^b^ Smoking status: (1) never smokers (<100 cigarettes), (2) former smokers (≥100 cigarettes, not current), (3) current smokers (≥100 cigarettes, actively smoking).

^c^ Drinking status: (1) never drinkers, (2) former drinkers, (3) mild drinkers (current, below moderate criteria), (4) moderate drinkers (women: ≥2 drinks/day; men: ≥3 drinks/day, or 2–4 binge days/month), (5) heavy drinkers (women: ≥3 drinks/day; men: ≥4 drinks/day, or ≥5 binge days/month).

^d^ Prediabetes diagnostic criteria: (1) clinical assessment, (2) HbA1c 5.7-6.4%, (3) FPG 5.6-6.9 mmol/L, (4) OGTT two-hour glucose 7.8-10.9 mmol/L. Diabetes diagnostic criteria: (1) medical diagnosis, (2) HbA1c >6.5%, (3) FPG ≥7.0 mmol/L, (4) random glucose ≥11.1 mmol/L, (5) OGTT two-hour glucose ≥11.1 mmol/L.

^e^ Hypertension definition: (1) SBP ≥140 mmHg, (2) DBP ≥90 mmHg, (3) physician diagnosis, (4) current antihypertensive medication use.

^f^ Hyperlipidemia definition: (1) triglycerides ≥150 mg/dL, (2) total cholesterol ≥200 mg/dL, (3) LDL-cholesterol ≥130 mg/dL, (4) HDL-cholesterol <40 mg/dL (men) or <50 mg/dL (women), (5) lipid-lowering medication use.

**Abbreviations:** GNRI, geriatric nutritional risk index; PIR, poverty income ratio; MET, metabolic equivalent; SBP, systolic blood pressure; DBP, diastolic blood pressure; BMI, body mass index; eGFR, estimated glomerular filtration rate; CI, confidence interval; HbA1c, glycated hemoglobin; FPG, fasting plasma glucose; OGTT, oral glucose tolerance test; LDL, low-density lipoprotein; HDL, high-density lipoprotein.

**Table S2. Subgroup analysis of association between GNRI quartiles and stroke**

| **Subgroup variable** | **Subgroup category** | **OR (95% CI)** | | | **Level-specific interaction P-value** | **Overall interaction P-value** |
| --- | --- | --- | --- | --- | --- | --- |
|  |  | **Q2 vs. Q1** | **Q3 vs. Q1** | **Q4 vs. Q1** |  |  |
| **Age (years)** | | | | | | 0.02* |
|  | <75 | 0.86 (0.71-1.04) | 0.72 (0.57-0.90) | 0.71 (0.55-0.90) | - |  |
|  | ≥75 | 0.70 (0.56-0.87) | 0.97 (0.76-1.24) | 0.62 (0.44-0.85) | 0.24, 0.04, 0.71 |  |
| **Sex** | | | | | | 0.36 |
|  | Male | 0.88 (0.71-1.09) | 0.91 (0.72-1.15) | 0.75 (0.57-0.97) | - |  |
|  | Female | 0.72 (0.59-0.88) | 0.72 (0.56-0.91) | 0.61 (0.45-0.82) | 0.14, 0.13, 0.28 |  |
| **Race/ethnicity** | | | | | | 0.03* |
|  | Non-white | 0.78 (0.64-0.96) | 0.62 (0.48-0.80) | 0.70 (0.53-0.93) | - |  |
|  | White | 0.78 (0.64-0.95) | 0.94 (0.76-1.17) | 0.62 (0.47-0.81) | 0.67, 0.06, 0.19 |  |
| **Smoking status** | | | | | | 0.75 |
|  | Never | 0.86 (0.69-1.08) | 0.93 (0.72-1.20) | 0.79 (0.58-1.06) | - |  |
|  | Former | 0.69 (0.55-0.86) | 0.69 (0.53-0.89) | 0.57 (0.42-0.76) | 0.25, 0.16, 0.20 |  |
|  | Current | 0.94 (0.65-1.36) | 0.93 (0.60-1.43) | 0.71 (0.41-1.19) | 0.75, 0.90, 0.76 |  |
| **Drinking status** | | | | | | 0.008* |
|  | Never | 1.00 (0.72-1.40) | 1.18 (0.81-1.73) | 0.98 (0.61-1.53) | - |  |
|  | Former | 0.89 (0.70-1.14) | 0.97 (0.74-1.28) | 0.76 (0.55-1.05) | 0.77, 0.47, 0.42 |  |
|  | Current | 0.67 (0.54-0.82) | 0.60 (0.46-0.77) | 0.54 (0.40-0.71) | 0.04, 0.001, 0.01 |  |
| **eGFR (ml/min/1.73 m^2^)** | | | | | | 0.52 |
|  | Low (<60) | 0.83 (0.66-1.03) | 0.76 (0.58-0.99) | 0.59 (0.42-0.82) | - |  |
|  | Moderate (60-89) | 0.81 (0.65-1.00) | 0.87 (0.68-1.11) | 0.69 (0.52-0.91) | 0.86, 0.48, 0.53 |  |
|  | High (≥90) | 0.56 (0.36-0.86) | 0.62 (0.37-1.00) | 0.67 (0.40-1.10) | 0.10, 0.42, 0.71 |  |

**Notes:** All values are presented as OR with 95% CI. The overall interaction P-value, derived from the likelihood ratio test, assesses the significance of the interaction between GNRI quartiles and the subgroup variable, with * indicating P < 0.05 (significant interaction). The level-specific interaction P-value corresponds to the interaction term (GNRI quartile × subgroup category) in the logistic regression model and is reported only for non-reference categories; reference categories (the first level of each subgroup variable) are denoted by “-”. For subgroup variables with multiple categories, the three P-values in the level-specific interaction P-value column correspond to the interaction terms between each GNRI quartile (Q2, Q3, Q4) and the specific subgroup category, presented in the order of Q2×category, Q3×category, and Q4×category interactions. For binary subgroup variables (age, sex, race/ethnicity), the level-specific and overall interaction P-values reflect the same interaction effect. All models were adjusted for age, sex, race/ethnicity, PIR, education level, METs/week, BMI, smoking status, drinking status, eGFR, glucose metabolism state, hypertension, and hyperlipidemia, excluding the stratifying variable.

**Abbreviations:** GNRI, geriatric nutritional risk index; OR, odds ratio; CI, confidence interval; eGFR, estimated glomerular filtration rate; PIR, poverty income ratio; MET, metabolic equivalent of task; BMI, body mass index.

**Table S3. Association between GNRI (z-score) and stroke prevalence stratified by age groups**

| **Age Group** | **Model** | **OR (95% CI)** | **P-value** |
| --- | --- | --- | --- |
| **60-69 years** |  |  |  |
|  | Model 1 | 0.78 (0.72, 0.85) | <0.001 |
|  | Model 2 | 0.77 (0.71, 0.84) | <0.001 |
|  | Model 3 | 0.87 (0.79, 0.95) | 0.003 |
| **≥70 years** |  |  |  |
|  | Model 1 | 0.84 (0.79, 0.90) | <0.001 |
|  | Model 2 | 0.86 (0.80, 0.92) | <0.001 |
|  | Model 3 | 0.89 (0.83, 0.96) | 0.002 |

**Model 1:** Non-adjusted.

**Model 2:** Adjusted for age and sex.

**Model 3:** Adjusted for age, sex, race/ethnicity, PIR, education level, BMI, smoking status, drinking status, glucose metabolism state, hypertension, hyperlipidemia, METs/week, and eGFR.

**Abbreviations:** GNRI, geriatric nutritional risk index; OR, odds ratio; CI, confidence interval; PIR, poverty income ratio; BMI, body mass index; MET, metabolic equivalent; eGFR, estimated glomerular filtration rate.

**Table S4. Association between GNRI and stroke prevalence in participants aged ≥65 years**

| **GNRI** | **OR (95% CI)** | | |
| --- | --- | --- | --- |
|  | **Model 1** | **Model 2** | **Model 3** |
| **Continuous (per one SD)** | 0.84 (0.79, 0.89) | 0.85 (0.80, 0.91) | 0.90 (0.85, 0.96) |
| **Quartiles** |  |  |  |
| Q1 | Reference | Reference | Reference |
| Q2 | 0.72 (0.61, 0.85) | 0.73 (0.62, 0.87) | 0.81 (0.68, 0.97) |
| Q3 | 0.66 (0.56, 0.79) | 0.68 (0.57, 0.81) | 0.76 (0.63, 0.91) |
| Q4 | 0.69 (0.58, 0.82) | 0.72 (0.61, 0.86) | 0.82 (0.69, 0.99) |
| **P for trend** | <0.001 | <0.001 | <0.05 |

**Model 1:** Non-adjusted.
**Model 2:** Adjusted for age and sex.
**Model 3:** Adjusted for age, sex, race/ethnicity, PIR, education level, BMI, smoking status, drinking status, glucose metabolism state, hypertension, hyperlipidemia, METs/week, and eGFR.
**Abbreviations:** GNRI, geriatric nutritional risk index; OR, odds ratio; CI, confidence interval; SD, standard deviation; PIR, poverty income ratio; BMI, body mass index; MET, metabolic equivalent; eGFR, estimated glomerular filtration rate.

**Table S5. Sensitivity analysis of association between GNRI and stroke with and without BMI adjustment**

| **Adjustment Strategy** | **OR (95% CI)** |
| --- | --- |
| **With BMI Adjustment** |  |
| Continuous (per SD increase) | 0.880 (0.832–0.932) |
| Q1 | Reference |
| Q2 | 0.809 (0.690–0.949) |
| Q3 | 0.737 (0.626–0.868) |
| Q4 | 0.770 (0.654–0.907) |
| **Without BMI Adjustment** |  |
| Continuous (per SD increase) | 0.882 (0.833–0.933) |
| Q1 | Reference |
| Q2 | 0.810 (0.691–0.950) |
| Q3 | 0.740 (0.628–0.871) |
| Q4 | 0.776 (0.660–0.913) |

**Notes:** All values are presented as odds ratios (ORs) with 95% confidence intervals (CIs), calculated using survey-weighted logistic regression models for the fully adjusted model. The “With BMI Adjustment” results correspond to Model 3 in Table 2, adjusted for age, sex, race/ethnicity, PIR, education level, BMI, smoking status, drinking status, glucose metabolism state, hypertension, hyperlipidemia, METs/week, and eGFR. The “Without BMI Adjustment” results replicate Model 3 but exclude BMI as a covariate.

**Abbreviations:** GNRI, geriatric nutritional risk index; BMI, body mass index; OR, odds ratio; CI, confidence interval; SD, standard deviation; PIR, poverty income ratio; MET, metabolic equivalent; eGFR, estimated glomerular filtration rate.


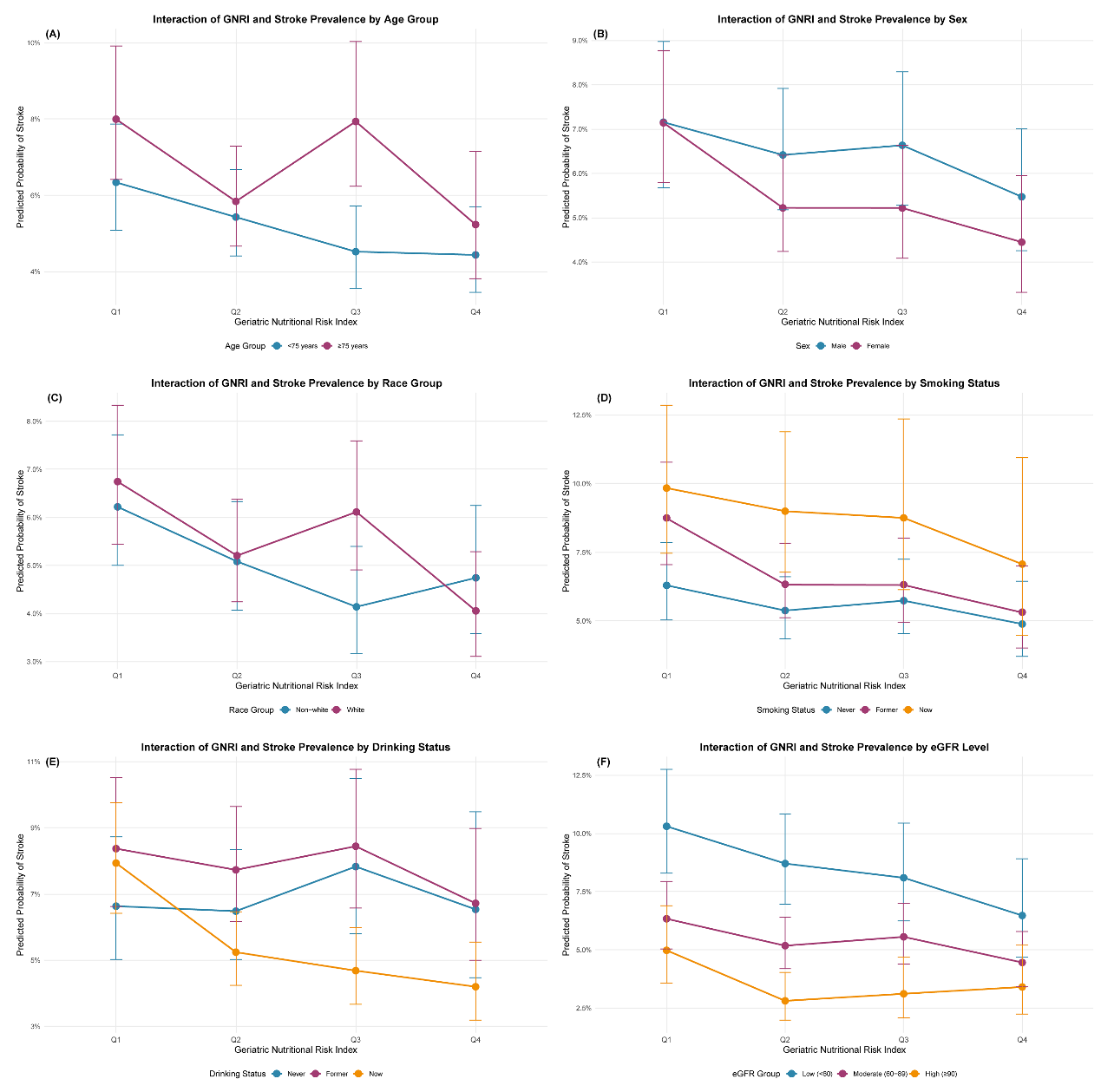


**Figure S1.** Interaction analysis of GNRI quartiles and stroke probability across multiple subgroups.

**Notes:** This figure depicts logistic regression models assessing the relationship between GNRI quartiles and stroke probability across subgroups defined by (A) age, (B) sex, (C) race/ethnicity, (D) smoking status, (E) drinking status, and (F) eGFR, with adjustments for confounders including age, sex, race/ethnicity, PIR, education level, BMI, smoking status, drinking status, glucose metabolism state, hypertension, hyperlipidemia, METs/week, and eGFR. Colors indicate subgroups within each variable, with distinct hues for each level. Short horizontal lines show 95% CIs for predicted probabilities.

**Abbreviations:** GNRI, geriatric nutritional risk index; eGFR, estimated glomerular filtration rate; PIR, poverty income ratio; BMI, body mass index; MET, metabolic equivalent; CI, confidence interval.

**Supplementary Text 1. Detailed notes and model specifications for Table 3**

Notes: All values are presented as OR with 95% CI. The overall interaction P-value, derived from the likelihood ratio test, assesses the significance of the interaction between GNRI and the subgroup variable. The level-specific interaction P-value corresponds to the interaction term (GNRI × subgroup category) in the logistic regression model and is reported only for non-reference categories; reference categories (the first level of each subgroup variable) are denoted by “-”. For binary subgroup variables (age, sex, race/ethnicity), the level-specific and overall interaction P-values reflect the same interaction effect. All models were adjusted for age, sex, race/ethnicity, PIR, education level, METs/week, BMI, smoking status, drinking status, eGFR, glucose metabolism state, hypertension, and hyperlipidemia, excluding the stratifying variable.
